# Role of noncoding RNA in the pathophysiology and treatment of intrauterine adhesion

**DOI:** 10.3389/fgene.2022.948628

**Published:** 2022-10-28

**Authors:** Hui-Dong Liu, Shao-Wei Wang

**Affiliations:** ^1^ Department of Gynecology and Obstetrics, Beijing Hospital, National Center of Gerontology, Institute of Geriatric Medicine, Chinese Academy of Medical Sciences, Beijing, China; ^2^ Graduate School of Peking Union Medical College, Peking Union Medical College, Chinese Academy of Medical Sciences, Beijing, China

**Keywords:** intrauterine adhesion, noncoding-RNA, microRNA, mesenchymal stem cells, exosome

## Abstract

Intrauterine adhesion (IUA) is one of the most common diseases of the reproductive system in women. It is often accompanied by serious clinical problems that damage reproductive function, such as menstrual disorder, infertility, or recurrent abortion. The clinical effect of routine treatment is not ideal, and the postoperative recurrence rate is still very high. Therefore, exploring the pathological mechanism of IUA and finding new strategies for the effective prevention and treatment of IUA are needed. The main pathological mechanism of IUA is endometrial fibrosis and scar formation. Noncoding RNA (ncRNA) plays an important role in the fibrosis process, which is one of the latest research advances in the pathophysiology of IUA. Moreover, the exosomal miRNAs derived from mesenchymal stem cells can be used to improve IUA. This paper reviewed the role of ncRNAs in IUA pathogenesis, summarized the core pathways of endometrial fibrosis regulated by ncRNAs, and finally introduced the potential of ncRNAs as a therapeutic target.

## Introduction

Intrauterine adhesion (IUA) is the fibrosis of damaged tissue in the basal layer of the endometrium, resulting in the adhesion of the uterine cavity and/or cervical lumen. IUA occurs secondary to endometrial injury, including trauma, curettage, infection, and congenital uterine malformation. Clinical manifestations include amenorrhea, oligomenorrhea, infertility, or recurrent abortion (Kelleher et al., 2018), which seriously affect women’s health and fertility. In recent years, the increase in intrauterine surgeries and hysteroscopy in pregnant women have resulted in a considerable increase in the incidence and diagnostic rates of IUA (Chen et al., 2017a). Transcervical resection of adhesions (TRCA) is the current method for IUA treatment. Hysteroscopy is considered the gold standard for IUA diagnosis and treatment (Vitale et al., 2022). Direct visualization of the uterine cavity allows the dissolution of adhesions by mechanical or electrosurgical energy (Vitale et al., 2020). Common means to prevent IUA recurrence after operation include the insertion of copper intrauterine device and the placement of a balloon catheter in the intrauterine cavity (Lin et al., 2015; Shi et al., 2019). In addition, the intrauterine infusion of self-cross-linked hyaluronic acid (HAG) can prevent adhesion recurrence and remarkably improve the postoperative pregnancy rate (Vitale et al., 2022). Notably, IUA occurs after blind curettage for the retention of pregnancy products (RPOC) (Vitale et al., 2021a). However, hysteroscopic resection of RPOC may replace blind curettage in RPOC treatment and therefore reduce the risk of IUA formation (Vitale et al., 2021b). Although the rational application of hysteroscopy and some postoperative strategies can normalize the uterine cavity morphology to varying degrees and even increase or restore the menstrual flow of some patients, many problems in clinical application, including postoperative adhesion recurrence and infertility, need to be solved (Xin et al., 2019). The recurrence rate of IUAs after operation is 62.5% (Chen et al., 2017b), and the overall pregnancy rate is only 42.8%–66.1% (Chen et al., 2017a). Therefore, exploring the pathological mechanism of IUA and finding new strategies for the effective prevention and treatment of IUA are crucial.

Noncoding RNAs (ncRNAs) refer to functional RNAs that are transcribed from DNA and have a structure similar to mRNA but are not translated into proteins (Beermann et al., 2016). A total of 8801 small ncRNAs (sncRNA, <30 NT) and 9640 long ncRNAs (lncRNAs, >200 NT) have been identified in the human genome (Qureshi and Mehler, 2012). NcRNAs can be divided into sncRNA, lncRNA, and circular ncRNA (circRNA) according to their length and shape. SncRNAs include miRNAs, snoRNAs, snRNAs, siRNAs, tRNAs, and piRNAs (Cech and Steitz, 2014). Among them miRNAs, are the most widely studied. Functional studies have shown that ncRNA plays an important role in mRNA translation, RNA processing, transposon inhibition, germline stability maintenance, gene expression regulation, chromatin modification, and silencing (Qureshi and Mehler, 2012; Beermann et al., 2016). Moreover, ncRNA has important regulatory functions in many biological processes, such as cell proliferation, adhesion, apoptosis, angiogenesis, and migration (Alexander et al., 2010). Notably, ncRNAs are extremely important in IUA occurrence and development. Abnormal ncRNA expression is likely to provide a theoretical basis for the early diagnosis and disease evaluation of IUA and provide a potential target for IUA treatment. One of the latest advances in IUA pathophysiology is to clarify the role of miRNAs and other ncRNAs, which provides an opportunity to develop new therapeutic strategies. In this review, we focus on the role of ncRNA in IUA pathogenesis and their potential as disease markers and therapeutic targets.

## Role of ncRNAs in the pathophysiology of intrauterine adhesion

The main pathological changes in IUA are endometrial fibrosis and scar formation (Liu et al., 2019a). Many factors related to tissue and organ fibroses, such as transforming growth factor-β1 (TGF-β1), connective tissue growth factor (CTGF), metalloproteinase-9 (MMP-9), and Smad3 (Xiao et al., 2017), are closely related to the pathogenesis of IUA. TGF-β1 is a cytokine that promotes extracellular matrix (ECM) secretion and induces epithelial–mesenchymal transformation (EMT) (Hu et al., 2018). It is considered a key driver for the development of fibrotic diseases (Hu et al., 2018). TGF-β1 is released by platelets at the injury site and is considerably increased in the endometrium of patients with IUA (Zhou et al., 2018). After TGF-β1 is released, TGF-β1 binds to type II TGF-β receptors (TGFBR2) on the surface of target cells, activates type I TGF-β receptors (TGFBR1), and forms complexes. Next, the complexes induce the phosphorylation of Smad2 and Smad3, which then forms a heterogeneous complex with Smad4 and binds to the DNA sequence of genes involved in the nuclear regulation of fibrosis (Hu et al., 2018). This process triggers a series of downstream signaling pathways and promotes the occurrence of fibrosis. Other transcription factors, such as angiotensinⅡ (AngⅡ), MMP-9,integrin ανβ3, NF-κB, CTGF-2, and a series of inflammatory factors or fibrosis-related factors, promote IUA through TGF-β1 (Xiao et al., 2017).

**FIGURE 1:**
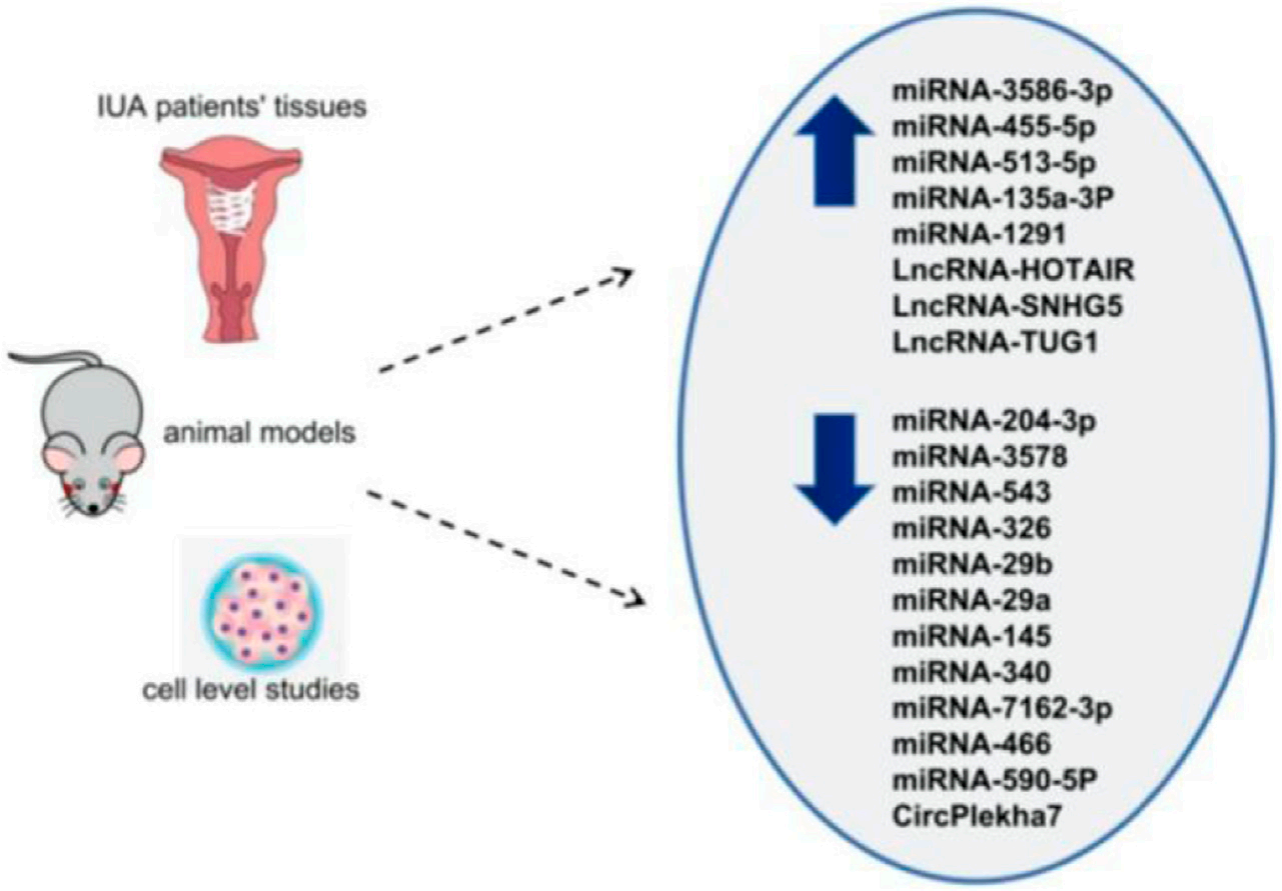
Differential expression of ncRNAs in IUA (upward arrow indicates high expression, downward arrow indicates low expression).

The three main core signaling pathways involved in endometrial fibrosis include the Wnt/β-catenin, PI3K-Akt/NF-κB, and TGF-β1/Smad signaling pathways. NcRNAs mostly target these pathways and participate in the regulation of IUA fibrosis. Table 1 displays all the ncRNAs currently reported to be involved in IUA pathophysiology. These studies included animal models, *in vitro* cell-level studies, and the evaluation of ncRNA expression in tissue samples from patients (Figure 1).

**TABLE 1:** ncRNAs involved in IUA pathophysiology.

MiRNA	Target genes	Signaling pathway	Reference
miR-543	*CDH2*, *COL16A1*, *MAPK*	Wnt/β-catenin	Liu et al. (2016); Wang et al. (2021); Liu et al. (2021a)
miR-513-5p	*ADAM-9*		Liu et al. (2016)
miR-135a-3p	*LOX*		Liu et al. (2016)
miR-326	*TGF-β1*	TGF-β/Smad3	Ning et al. (2018)
miR-29a		TGF-β/Smad3	Tan et al. (2020)
miR-29b	*Sp1*	TGF-β/Smad3	Li et al. (2016)
miR-145	*TGFBR2*	TGF-β/Smad2/Smad3	Xu et al. (2020)
miR-340	*TGFBR1*	TGF-β/Smad3	Xiao et al. (2019)
miR-1291	*ArhGAP29*	RhoA/ROCK1	Xu et al. (2017)
miR-466	*NUS1*	AKT/NF-κB	Liu et al. (2019c)
lncR-HOTAIR		TGF-β/Smad3	Wu et al. (2020)
lncR-SNHG5	*FOXF2*	Wnt/β-catenin	Liu et al. (2020c)
lncR-TUG1	*miR-590-5p/Fasl*		Ai et al. (2020)
circPlekha7	*miR-207*		Xie et al. (2020)

### MiRNA

MiRNA is a noncoding single-stranded RNA molecule with 20–23 nucleotides encoded by endogenous genes that widely exist in eukaryotes. It controls post-transcriptional gene expression by inhibiting translation or promoting mRNA degradation in the cytoplasm (Treiber et al., 2019). MiRNAs play an important role in all aspects of tissue repair, including inflammation, angiogenesis, fibroblast function, apoptosis, and collagen deposition (Rutnam et al., 2013). Notably, the abnormal expression or dysfunction of miRNAs plays an important role in various types of tissue fibrosis, such as heart (Cao et al., 2017), kidney (Liu et al., 2019b), liver (Calvente et al., 2019), and lung fibroses (Wei et al., 2019). Moreover, increasing studies have shown that miRNA imbalance is involved in IUA. The specific regulatory mechanisms will be elaborated in detail.

The researchers wanted to study the role of miRNAs in IUA. An IUA cell model was constructed by TGF-β1, and a Smad3 inhibitor (SIS3) was used to affect TGF-β1. The differential expression of miRNAs in the two groups were analyzed by high-throughput sequencing (Liu et al., 2020a). The results showed that the TGF-β1 + SIS3 group had 235 considerably upregulated differentially expressed miRNAs (DEMs) and 530 substantially downregulated DEMs compared with the TGF-β1 group. The Gene Ontology (GO) analysis of the 756 DEMs showed that the gene function focused on cell differentiation, regulation, and apoptosis. Kyoto Encyclopedia of Genes and Genomes (KEGG) analysis showed that these miRNAs were enriched in the MAPK and PI3K-Akt pathways. In addition, the experimental group also showed that miRNA-3586-3p and miRNA-455-5p overexpression could promote cell proliferation, inhibit apoptosis, and aggravate IUA pathogenesis. On the contrary, miRNA-204-3p and miRNA-3578 overexpression can inhibit cell behavior and IUA progression. The results suggest that these four miRNAs may participate in the pathological process of IUA and hopefully contribute to the further diagnosis and treatment of IUA, which provides an opportunity to explore the molecular mechanism of IUA from a new perspective. Finally, the authors proposed that miRNA-204 may exert a fine-tuning regulation of the synergistic transduction of PI3K/AKT/FAK mediators critical in vasculogenic mimicry formation. The PI3K/AKT pathway stood out from the results of the enrichment analysis and mechanism study; thus, this pathway may play an important role in IUA occurrence and development and is worthy of attention in subsequent studies.

Another study comprehensively analyzed the miRNA and mRNA expression profiles of eight patients with IUA and adjacent normal endometrium and constructed a transcriptome regulation network (Zong et al., 2021). A total of 72 DEMs (5 upregulated and 67 downregulated) were identified. GO enrichment analysis showed that the DEMs’ target genes were involved in angiogenesis regulation, MAPK activation, the negative regulation of cell migration, the positive regulation of epithelial cell proliferation, the regulation of typical Wnt signal pathway, and positivethe regulation of cell proliferation. The KEGG pathways that involved the DEMs include the RAS signal transduction pathway, Hippo signal transduction pathway, MAPK signal pathway, PI3K/Akt signal transduction pathway, gap junction, and Wnt signal transduction pathway.

Interestingly, the previous study also verified the involvement of DEMs in the PI3K–Akt pathway. Thus, abnormalities in this pathway may compromise endometrial development. For instance, the PI3K/Akt signal transduction pathway is considered to be involved in endometrial regeneration induced by granulocyte macrophage colony-stimulating factor (CSF) therapy (Liu et al., 2020b). The rapid activation of the PI3K/Akt signal pathway by growth factors and estrogen is involved in the migration of normal endometrial stromal cells (Gentilini et al., 2007). Therefore, the PI3K/Akt signal pathway has gradually become the star signal pathway in the pathological mechanism of IUA. The ncRNAs involved in regulating this signaling pathway and thus affecting IUA occurrence and development were determined. Liu et al. showed that miRNA-466 can target NUS1, negatively regulate the Akt pathway, and finally inhibit IUA (Liu et al., 2019c). This group verified that miRNA-466 overexpression could inhibit cell proliferation, migration, and invasion; reduce the cell cycle process; and inhibit EMT. An analysis of the expression level of Akt pathway-related genes and NF-κB activity clearly showed that miRNA-466 inactivated the Akt/NF-κB pathway by downregulating NUS1 and thus affecting the IUA process. Wang et al. also proposed that NF-κB is a new pathogenic factor of Asherman syndrome, which provides a new idea for the prevention and treatment of IUA in patients (Wang et al., 2017).

Current studies on the PI3K/Akt signal pathway in IUA’s pathological mechanism are limited, and only miRNA-466 has been explored in related ncRNA. However, this study is just the beginning, and further research is needed to explore which ncRNAs are involved in regulating the PI3K/Akt signal pathway and thus affecting IUA pathology.

Another high-throughput sequencing study of IUA was performed through the high-throughput microarray analysis of miRNAs differentially expressed in the three cases of severe IUA and three cases of normal endometrium, and 26 DEMs were obtained (Liu et al., 2016). They also verified the clinical samples of three obviously maladjusted DEMs and their corresponding target genes by real-time quantitative polymerase chain reaction. The results showed that miRNA-543 was downregulated in IUA, which could negatively regulate N-cadherin (CDH2) and inhibit EMT. The biological function of EMT is to produce fibroblasts and repair the tissue damage caused by trauma or inflammatory reaction. EMT stops under normal circumstances but persists in the activation of inflammatory response, which eventually leads to organ fibrosis. Therefore, during IUA formation, the EMT of CDH2 may be negatively regulated by miRNA-543 to produce fibroblasts. MiRNA-543 can also negatively regulate collagen 16A1 (COL16A1), which plays an important role in cell adhesion as a structural protein of the ECM. Thus, miRNA-543 can affect IUA formation by regulating the structural proteins of COL16A1 and ECM. MiRNA-513-5p is upregulated in IUA and negatively regulates ADAM-9. ADAM-9 belongs to the transmembrane disintegrin-containing metalloproteinase (ADAM) family, which is involved in extraprotein domain shedding and cell–cell and cell–matrix interactions (Buranaphatthana et al., 2021). Mauch et al. (2010) studied the role of ADAM9 in the process of excision wound healing and found that animals lacking ADAM-9 had faster wound repair compared with the control group. Therefore, in IUA pathogenesis, miRNA-513a-5p negatively regulates ADAM-9, which may be a mechanism to regulate cell migration and accelerate endometrial repair. MiRNA-135a-3p is upregulated in IUA and negatively regulates lysine oxidase (LOX). LOX is a copper-dependent amine oxidase that plays a key role in the fibrosis process by crosslinking ECM protein, collagen, and elastin (Tadmor et al., 2013). Thus, the expression of *LOX* gene family members is increased in disorders associated with increased fibrosis. In an animal model of liver fibrosis (Perepelyuk et al., 2013), increased tissue stiffness precedes the activation of fibroblastic cells and the accumulation of collagen, suggesting that such early mechanical changes may be sufficient to trigger the contraction cascade. Collagen crosslinking catalyzed by LOX enzymes is one possible factor responsible for the early structural changes and tissue stiffening seen in these conditions. However, the study shows that miRNA-135a-3p negatively regulates LOX and downregulates LOX expression in IUA, which suggests that the lack of LOX may have an effect on IUA formation. In this study, three miRNAs involved in the IUA process were explored through high-throughput sequencing and mechanism verification, and the targeted genes were identified. MiRNA-543 can inhibit endometrial fibrosis and EMT, miRNA-513a-5p negatively regulates the endometrial repair of ADAM-9 tissue, and the high miRNA-135A-3p expression in IUA inhibits the role of *LOX*, a target gene that plays a key role in the fibrosis process.

Through the whole-gene analysis of the miRNA transcriptome, we can fully understand the differentially expressed ncRNAs and find their target genes and corresponding signal pathways through GO and KEGG enrichment analyses. Studying the mechanism of DEMs in IUA is of great importance. Moreover, exploring the mechanism of action of specific ncRNAs in depth is necessary to better understand the role of ncRNAs in the pathological mechanism of IUA.

One study showed that miRNA-326 can directly target the negative regulation of the TGF-β/Smad3 pathway to inhibit endometrial fibrosis (Ning et al., 2018). The research also measured the expression level of miRNA-326 and the mRNA expression levels of fibrosis marker genes (alpha smooth muscle actin [a-SMA], collagen 1A1 [COL1A1], and fibronectin [FN]) in 30 IUA endometrial tissues. The results showed that the mRNA expression levels of a-SMA, COL1A1, and FN were negatively correlated with the expression level of miRNA-326. This result suggests that miRNA-326 may be an effective biomarker for the prognosis of patients with IUA. TGF- β1 can inhibit miRNA-29 expression (Horita et al., 2021). On the contrary, miRNA-29 overexpression can reduce TGF-β1 to reduce Smad3 activity and achieve \ anti-fibrosis (Horita et al., 2021). This finding indicates that miRNA-29 may be a potential target for anti-fibrosis therapy. The miRNA-29 family includes miR-29a, miR-29b, and miR-29c (Horita et al., 2021). Among these subtypes, the target gene of miRNA-29a is most related to fibrosis (Alizadeh et al., 2019). A study has shown that miRNA-29a overexpression in the exosomes of bone marrow mesenchymal stem cells (BMSCs) can reduce the expression of a-SMA, collagen I, Smad2, and Smad3 in the cell model (Tan et al., 2020). The study confirmed that miR-29a as an anti-fibrosis gene can play an effective role in IUA treatment. Li et al. proposed that miRNA-29 b blocks SP1-TGF- β/Smad3-CTGF pathway and inhibits endometrial fibrosis (Li et al., 2016). The group verified that miRNA-29b is remarkably underexpressed in fibrotic endometrium, and the levels of COL1A1, a-SMA, and E-cadherin decrease after miR-29b overexpression. These findings suggest that miR-29b can prevent ECM overdeposition and EMT after endometrial injury. In the mechanism study, miR-29b was obtained to block Sp1–Smad3 interaction by directly targeting the transcription factor Sp1, inhibiting TGF-β/Smad3-mediated endometrial fibrosis. MiRNA-145 was verified to be lowly expressed in IUA and can target TGFBR2 to regulate the TGF-β/Smad2/Smad3 pathway to achieve anti-fibrosis (Xu et al., 2020). In the experiment, the expression of fibrosis marker genes increased in TGF-β1-induced endometrial stromal cells, and miRNA-145 overexpression reversed the result. The double-luciferase reporter gene system determined that miR-145 directly targets TGFβR2. Moreover, miRNA-145 overexpression decreases the expression of TGFβR2, TGF-β1, and Smad2/3. MiRNA-145 can inhibit fibrotic progression *via* TGF-β/Smad2/Smad3. Another miRNA targeting the TGF-β receptor gene is miRNA-340 (Xiao et al., 2019). This study showed that miRNA-340 can target TGF-β receptor 1 (TGFβR1) to inhibit TGF-β1 expression to induce a-SMA and COL1A1; thus, miRNA-340 plays an anti-fibrosis role.

The above studies are focused on the miRNAs involved in the TGF-β1/Smad signal pathway. This pathway has been recognized as the core pathway involved in endometrial fibrosis in IUA, and multiple miRNAs play a role in this pathway.

MiRNA-326, miRNA-29a/b, MiRNA-145, and miRNA-340 are all remarkably underexpressed in IUA and play an anti-fibrosis role by acting on the TGF-β1/Smad signal pathway. The target genes of miRNA-326, miRNA-29b, miRNA-145, and miRNA-340 are *TGF-β1*, *Sp1*, *TGFBR2*, and *TGFBR1*, respectively. More ncRNAs related to the TGF-β1/Smad signal pathway are expected to be excavated in future in-depth studies to have a more comprehensive understanding of the mechanism of this pathway in IUA.

One study pointed out that miRNA-1291 and its target gene do not directly regulate the TGF- β/Smad3 pathway but act on the TGF-β1 downstream target of signal transduction, RhoA (Xu et al., 2017). RhoA with GTPase activity belongs to a subgroup member of the small G protein superfamily (Nguyen et al., 2018). It is the intermediary of a variety of signal proteins in cell surface receptor siblings (Nguyen et al., 2018). In cells undergoing EMT, TGF-β1 through non-standard TGF-β1 signal and the signal pathway of complement Smad, which is sent by type II receptor. TGFβR2 binding ligand directly phosphorylates cell polarity regulator segmentation defect 6 (PAR6) (Mitamura et al., 2018). Phosphorylated PAR6 recruits SMURF1 to target RhoA GTP at tight junction, resulting in junction dissolution and polarization migration (Mitamura et al., 2018). Previous studies have shown that RhoA and its effector ROCK are closely related to several fibrinogenic diseases in the lung (Bei et al., 2016), kidney (Jia et al., 2015), cardiovascular tissue (Gao et al., 2013), and liver (Zhang et al., 2016). Xu et al. showed that miRNA-1291 acts on ArhGAP2, and ArhGAP2 negatively regulates RhoA/ROCK1-EMT (Xu et al., 2017). MiRNA-1291 antagonist could reduce the expression of a-SMA, COL1A1, platelet-derived growth factor-BB and fibroblast growth factor 2. Masson trichrome staining showed that miRNA-1291 antagonist could remarkably reduce the degree of endometrial fibrosis. Therefore, miRNA-1291 antagonist can block the RhoA/ROCK1 pathway, reduce fibrosis, and improve IUA. MiRNA-1291, as a pro-fibrosis miRNA, is expected to become a new therapeutic target and biomarker for IUA.

The last core signal pathway of endometrial fibrosis is the Wnt/β-catenin signal pathway. The Wnt/β-catenin signal pathway is a common pathway involved in the regulation of a variety of diseases (Huang et al., 2019). Moreover, many studies have shown that this pathway is involved in regulating the phenotype of endometrial stromal cells (Zhou et al., 2019a; Lv et al., 2020). Wang et al. showed that miRNA-543 inhibits Wnt/β- Catenin pathway by targeting MAPK and therefore inhibits ECM protein level and the EMT process (Wang et al., 2021). This experiment verified that miRNA-543 is considerably lowly expressed in TGF-β-treated embryonic stem cells (ESCs), and its anti-fibrosis effect was verified functionally. Finally, the mechanism was explored, and the results showed that miRNA-543 inactivates the Wnt/β-catenin signal pathway by negatively regulating MAPK to improve IUA. Moreover, estrogen attenuates TGF-β1-induced EMT in IUA by activating the Wnt/β-catenin signaling pathway (Cao et al., 2020). MAPK, an extracellular signal-regulated kinase, is involved in a variety of cellular processes, such as cell proliferation, differentiation, and transcriptional regulation (Wang et al., 2020a). Notably, the enrichment analysis of the DEMs mentioned above is concentrated in the MAPK pathway (Liu et al., 2020a; Zong et al., 2021). From this finding, the MAPK/Wnt/β-catenin signal pathway is thought to play an important role in the pathological mechanism of IUA. Similarly, few studies on IUA have focused on this pathway; thus, more attention should be given to this pathway in future studies.

MiRNA-543 has been mentioned many times above that studying it with a new eye is difficult. Another study pointed out that miRNA-543 can directly and negatively regulate CDH2 (N-cadherin) to inhibit the degree of fibrosis, a W3reduce the collagen content of uterine adhesive tissue, and participate in IUA occurrence and development (Liu et al., 2021a). Multiple studies have pointed out the anti-fibrosis effect of miRNA-543, proving that miRNA-543 is expected to play a role in IUA treatment.

### LncRNA

LncRNA is a kind of ncRNA with a length greater than 200 NT (Morlando et al., 2015). Although lncRNAs do not have a protein-coding function, they participate in epigenetic regulation, transcription, and post-transcriptional modification, and are key regulatory factors in many pathological and physiological processes, including cell differentiation, proliferation, and apoptosis (Morlando et al., 2015). In addition, lncRNA can act as a competitive endogenous RNA (ceRNA) of miRNA and participate in a series of cellular pathways. For example, a study found that lncRNA-TUG1 acts as the ceRNA of miRNA-197-3p to regulate TUMS expression in colorectal cancer, mediating 5-fluorouracil resistance (Wang et al., 2019). Qian et al. (2019) found that lncRNA-ZEB1-AS1 regulates pulmonary fibrosis through competitive binding to miRNA-141-3p, promoting the EMT process. Increasing evidence showed that lncRNA is involved in some important organ fibrosis processes, including pulmonary fibrosis (Xu et al., 2019a), diabetic fibrosis (Ge et al., 2019), and cardiac fibrosis (Kenneweg et al., 2019). However, studies on the mechanism of lncRNA in IUA has just begun. It also involves several of the classical pathways of fibrosis mentioned above.

LncRNA-HOTAIR activates the TGF-β/Smad3 pathway and promotes endometrial fibrosis (Wu et al., 2020). This test proved that the expression level of lncRNA-HOTAIR in IUA is remarkably increased, and the fibrosis level is increased after lncRNA-HOTAIR overexpression, and *vice versa*. Whether other cytokines are involved in the regulation has not been further studied. Another study showed that lncRNA-SNHG5 regulates Wnt/β-catenin by targeting FOXF2 and participates in fibrosis (Liu et al., 2020c). This experiment verified that lncRNA-SNHG5 is highly expressed in the IUA cell model and positively regulates FOXF2, which verified that silencing lncRNA-SNHG5 could effectively block the Wnt/β-catenin pathway and inhibit the process of fibrosis. LncRNA can act as the ceRNA of miRNA and participate in a series of cellular pathways. LncRNA-TUG1 can increase TGF-β-induced inflammatory response and the EMT of human ESCs by competitively binding miRNA-590-5P(Ai et al., 2020). This research verified that LncRNA-TUG1 is considerably overexpressed in the endometrial tissues of patients with IUA and in the IUA cell model, and lncRNA-TUG1 knockout could substantially inhibit the EMT process and the secretion of inflammatory cytokines. Finally, lncRNA-TUG1/miRNA-590-5p/FasL was identified to be involved in IUA fibrosis. The related pathways that it targets have not been clarified yet. LncRNA-HOTAIR, lncRNA-SNHG5, and lncRNA-TUG1 act as pro-fibrosis genes in IUA occurrence and development, and the inhibition of their expression can inhibit the fibrosis process and thus play a therapeutic role. We can also appropriately speculate that the high expression of most lncRNAs in IUA plays a role in promoting fibrosis. Moreover, these lncRNAs are likely to act as ceRNAs by sponging the corresponding miRNAs. For example, lncRNA-TUG1 acts as ceRNA of miRNA-590-5p. However, whether LncRNA-HOTAIR and LncRNA-SNHG5 also act on miRNAs needs further study, and it is also crucial to identify other lncRNAs that may be involved in the pathological process of IUA.

A recent study established a complete lncRNA–miRNA–mRNA network in IUA based on ceRNA theory (Zhang et al., 2022a). This study identified 418 differentially expressed lncRNAs and 915 differentially expressed miRNAs. The target genes of differentially expressed miRNAs were predicted using TargetScan, and the intersection of target genes and DEGs was obtained. Finally, a network of upregulated ceRNAs was constructed. Some potential ncRNAs that may be involved in the pathological process of IUA were identified, such as ADIRF-AS1, LINC00632, DIO3OS, MBNL1-AS1, miR-326, miR-155-5p, miR-874-3p, miR-503-5p, and miR-149-5p. The roles of these lncRNAs in regulating the expression pattern and biological characteristics of miRNAs in IUA have not been extensively studied. However, this experiment made a macroanalysis of the ceRNA network diagram of IUA and screened out multiple differentially expressed lncRNA–miRNAs, providing important clues for the further study of ceRNA network in the future.

### CircRNA

CircRNA is a new kind of endogenous ncRNA. Different from traditional linear RNAs (miRNAs and lncRNAs), circRNAs are characterized by a closed ring structure formed by reverse splicing and have no 5′ N and 3′ U (Memczak et al., 2013). Their expression has high cell specificity, tissue specificity, and development specificity. CiRNA can be used as a miRNA sponge, a protein-binding molecule, and a transcription regulator (Cheng et al., 2019).

CircRNA acts as a sponge on miRNA and is therefore considered to have a negative regulatory effect on miRNA and make a remarkable contribution to the ceRNA network. CircRNA has roles in fibrosis, including cardiac fibrosis and liver fibrosis (Zhou et al., 2019b; Zhu et al., 2019). However, studies on IUA are few. CircPlekha7 overexpression can inhibit the expression levels of a-SMA, collagen I, and Smad3; inhibit the activity of ESCs; and promote apoptosis (Xie et al., 2020). The research also pointed out that circPlekha7 is a direct target of SIS3 and plays an anti-fibrosis role. In addition, the high expression of circPlekha7 can lead to the decreased expression level of miR-207. On the contrary, the expression level of miR-207 is considerably increased after the silencing of circPlekha7; therefore, circPlekha7 may be involved in fibrosis-related pathways in IUA by targeting miRNA or regulating its host genes. However, the specific regulatory mechanism needs more in-depth studies.

## Potential therapeutic role of ncRNA in intrauterine adhesion

**FIGURE 2:**
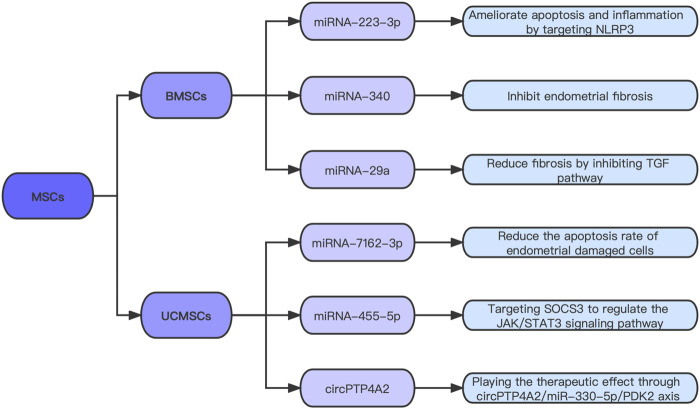
Exosomal ncRNAs candidates for IUA treatment.

Although the application of hysteroscopy plays a pivotal role in IUA diagnosis and treatment, and some postoperative physical defense measures have a certain improvement effect, the recurrence of adhesions is still worrying. Stem cell therapy has become a research hotspot in recent years. Mesenchymal stem cells (MSCs) are a kind of pluripotent stem cells that have the ability of self-renewal and multidirectional differentiation; therefore, they have become one of the most commonly used cell types in the regenerative medicine domain. In the field of IUA, many studies have also confirmed that stem cell therapy plays an important role in repairing damaged endometrium and improving IUA (Zheng et al., 2020; Min et al., 2021). However, the molecular mechanisms underlying the therapeutic effects of MSCs are not well understood. Which cytokines play a role in the treatment process remain to be further studied (Figure 2).

After confirming that umbilical cord mesenchymal stem cells (UCMSCs) can improve uterine appearance and morphological characteristics, a study analyzed the differential expression of miRNAs in the IUA model group and the MSCs treatment group (Zhang et al., 2022b). Compared with the model group, 55 miRNAs were up-regulated and 59 miRNAs were down-regulated in the treatment group. An interaction network of differentially expressed miRNAs and DEGs was established. This study revealed the interrelationship between miRNAs and mRNAs during endometrial injury and repair, and provided a new approach for MSCs to treat IUA. Sun et al. (2021) found that miR-455-5p could promote the proliferation and cell cycle progression of ESCs co-cultured with UCMSCs, and *in vivo* experiments verified that high expression of miR-455-5p could improve the therapeutic effect of UCMSCs on endometrial fibrosis. Mechanically, this experiment explored that miR-455-5p in MSCs promotes the repair of damaged endometrium by directly targeting SOCS3 to regulate the JAK/STAT3 signaling pathway. Zheng et al. (2022) found that circPTP4A2 expression is remarkably increased in UCMSCs, reduces the area of fibrosis, and increases the number of glands in IUA animal models by targeting miRNA-330-5p. This study explored the therapeutic effect of the circPTP4A2/miR-330-5P/PDK2 axis on IUA, which is expected to become another ncRNA target for the precise treatment of IUA.

These findings suggest that the application of ncRNAs in MSCs may provide a new idea for IUA treatment. In-depth study of the specific role of ncRNAs in the repair of endometrial fibrosis, the differentiation of their roles in anti-fibrosis or pro-fibrosis, and their appropriate application in MSC treatment will greatly improve the therapeutic effect against IUA. However, some safety problems exist in the clinical application of stem cell therapy, such as tumorigenicity and thrombosis (Sharpe et al., 2012; Jung et al., 2013). With the deepening of research, researchers found that the therapeutic effect of MSCs is mediated by their secreted exosomes (Xu et al., 2019b). Compared with traditional cells, the biological activity of exosomes is more stable and easy to preserve, the extraction process is easier to standardize, and the risk of tumorigenicity is unremarkable (Xu et al., 2019b). Therefore, it is expected to become a new strategy or cell-free therapy. Exosomes are small vesicles with a diameter of 30–150 nm (Tkach and Thery, 2016). Exosomes are composed of proteins, lipids, mRNA, and miRNA (Tkach and Thery, 2016). When exosomes are transported to target cells, they fuse with the plasma membrane of the target cells or are endocytosed into cells, releasing their contents and performing their functions (Tkach and Thery, 2016).

MSC-derived exosomes (MSC-Exos) have been considered to have great prospects as miRNA carriers in translational medicine(Nikfarjam et al., 2020). Studies have found that miRNA-133b transported by umbilical cord mesenchymal stem cell exosomes (UCMSC-Exos) promotes the proliferation, migration, and invasion of preeclampsia trophoblasts by inhibiting the glucocorticoid regulated kinase one gene (Wang et al., 2020b). MSC-Exos with miRNA-210 overexpression improve the protection of cardiac myocytes against stress *in vitro* and *in vivo* (Cheng et al., 2020). Therefore, whether a therapeutic effect can be achieved by regulating the expression level of the corresponding ncRNA in MSC-Exos in IUA treatment should be studied.

Xiao et al. (2019) verified that miRNA-340 expression is considerably low in IUA and high in BMSCs and their exosomes. Exosome inhibitors were used to verify that miRNA-340 is transported through exosomes (rather than the culture medium that removes exosomes). Subsequently, the results confirmed that BMSC-Exos with high miRNA-340 expression could remarkably promote endometrial recovery. Tan et al. (2020) found that BMSC-Exos overexpressing miRNA-29a can reduce the expression of fibrosis markers, such as a-SMA and collagen I. Another experiment showed that miRNA-7162-3p overexpression in UCMSC-Exos reduces the apoptosis rate of damaged endometrial cells (Shi et al., 2021). This result indicates that the UCMSC-Exos overexpressed by miRNA-7162-3p have great prospects in the cell-free treatment of endometrial injury. Liu et al. (2021b) found that BMSC-Exos are rich in miR-223-3p and can improve uterine injury. In this study, miR-223-3p expression in uterine endothelial progenitor cells (EPCs) was increased by BMSC-Exo treatment. Overexpressed miR-223-3p ameliorates the lipopolysaccharide (LPS)-induced apoptosis and inflammation of EPCs and promotes angiogenesis and IUA repair by directly targeting NLRP3. They concluded that BMSC-Exos improve LPS-induced IUA by delivering miR-223-3p to inhibit NLRP3 in a series of *in vitro* trials.

The above experiments showed that exosomes carry beneficial ncRNAs to the damaged endometrium and then release the ncRNAs to play a therapeutic role. Notably, exosomes carry ncRNAs by two ways. One way is that exosomes are rich in beneficial ncRNAs, which can be targeted by sequencing and experimental verification. The other is to transfect known beneficial ncRNAs into MSCs for high expression and extract exosomes to obtain exosomes with high ncRNA expression. Therefore, the exogenous release of beneficial ncRNA expression in MSC-Exos to promote endometrial recovery will become the focus of the cell-free treatment of IUA. However, studies on IUA treatment by exosome-derived ncRNA have just begun. What other anti-fibrosis ncRNAs are enriched in stem cell exosomes and whether these enriched ncRNAs have therapeutic effects on IUA need further investigation. Moreover, exosome therapy still has some limitations. Exosomes are in a static state and cannot reproduce in the body because of their cell-less structure. In addition, exosomes can be quickly cleared by host cells, resulting in a short half-life *in vivo* and limited therapeutic effect. Therefore, finding ways to improve the retention time of exosomes in the body is a research focus.

MSC-Exo can also be organically combined with scaffolds and other carriers to prolong its duration in the local tissue and enhance and optimize its curative effect. Collagen scaffold (CS), a nature biomaterial matrix, has been widely used in stem cell-loaded tissue regeneration owing to its good biocompatibility and controllable biodegradation (Xin et al., 2020). One study integrated CS with stem cell exosomes and verified that exosomes adhere closely, are distributed evenly on CS, and could continuously release exosomes *in vivo*, considerably improving the half-life of exosomes (Xin et al., 2020). *In vitro* and *in vivo* experiments confirmed that CS/Exos can promote endometrial repair and improve fertility. Moreover, miRNA microarray sequencing was performed on exosomes and stem cells in this experiment, which proved that miR-223-3p is a key component in exosomes that promotes endometrial repair. However, its specific mechanism of action needs further study. Lin et al. (2021) designed an injectable multifunctional microenvironment-protected exosome–hydrogel, which can continuously release exosomes and have anti-infection and microenvironmental protection properties, because it was formulated *via* Ag–S coordination endowing and dynamic coordination to fuse with adipose stem cell-derived exosomes. Other carrier materials include regenerated patches and nanocomposites. The integration of biological materials and stem cell exosomes could overcome some of the shortcomings of exosomes; provide a convenient, safe and non-invasive treatment method for repairing endometrium and restoring fertility; and can be applied clinically to benefit women.

## Summary

**FIGURE 3:**
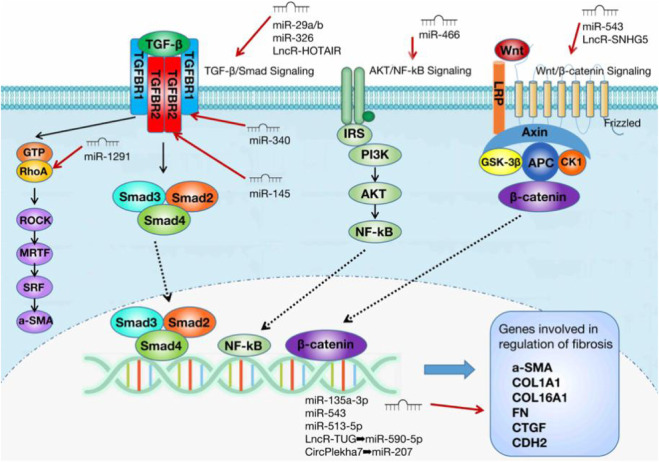
Three core signaling pathways of endometrial fibrosis involving ncRNAs.

This paper summarizes the research progress of ncRNAs in the pathological mechanism of IUA, and the three core signaling pathways of endometrial fibrosis involving ncRNAs: TGF-β/Smad3, Wnt/β-catenin, and AKT/NF-κB (Figure 3). It provides a solid foundation for further research on the molecular mechanism involved in IUA development. However, some key issues still need to be further explored. For example, more fibrosis-related ncRNAs need to be mined, some antifibrosis miRNAs’ target genes and related regulatory signaling pathways have not been clarified, and the specific regulatory roles of lncRNAs and circRNAs as ceRNAs need to be further studied.

In addition, it also introduced the potential therapeutic effects of ncRNAs from stem cell exosomes on IUA, and its combination with biological materials can better improve the therapeutic effect, which is likely to become an effective and safe cell-free therapy. At present, this treatment has been performed in animal models; however, many problems need to be solved and require further research prior to its clinical application.
