# Corrigendum: Role of non-coding RNA in the pathophysiology and treatment of intrauterine adhesion

**DOI:** 10.3389/fgene.2023.1141282

**Published:** 2023-01-26

**Authors:** Hui-Dong Liu, Shao-Wei Wang

**Affiliations:** ^1^ Department of Gynecology and Obstetrics, Beijing Hospital, National Center of Gerontology, Institute of Geriatric Medicine, Chinese Academy of Medical Sciences, Beijing, China; ^2^ Graduate School of Peking Union Medical College, Peking Union Medical College, Chinese Academy of Medical Sciences, Beijing, China

**Keywords:** intrauterine adhesion, non-coding-RNA, microRNA, mesenchymal stem cells, exosome

In the published article, there was an error in the **author affiliations**. They should be listed as follows:

“Hui-Dong Liu^1,2^ and Shao-Wei Wang^1,2,^*^”^

^“1^Department of Gynecology and Obstetrics, Beijing Hospital, National Center of Gerontology, Institute of Geriatric Medicine, Chinese Academy of Medical Sciences, Beijing, China”

^“2^Graduate School of Peking Union Medical College, Peking Union Medical College, Chinese Academy of Medical Sciences, Beijing, China”

The authors apologize for these error and state that this does not change the scientific conclusions of the article in any way. The original article has been updated.

